# Effects of interprofessional education for medical and nursing students: enablers, barriers and expectations for optimizing future interprofessional collaboration – a qualitative study

**DOI:** 10.1186/s12912-018-0279-x

**Published:** 2018-04-10

**Authors:** Sabine Homeyer, Wolfgang Hoffmann, Peter Hingst, Roman F. Oppermann, Adina Dreier-Wolfgramm

**Affiliations:** 1 Institute for Community Medicine, Department Epidemiology of Health Care and Community Health, University Medicine Greifswald, Ellernholzstr. 1-2, 17487 Greifswald, Germany; 2 Nursing Board, University Medicine Greifswald, Fleischmannstraße 8, 17475 Greifswald, Germany; 3 Department Nursing, Health and Administration, University of Applied Science Neubrandenburg, Brodaerstr. 2, 17033 Neubrandenburg, Germany

**Keywords:** Interprofessional education, Education research, Education, medical, graduate, Education, nursing, Qualitative research

## Abstract

**Background:**

To ensure high quality patient care an effective interprofessional collaboration between healthcare professionals is required. Interprofessional education (IPE) has a positive impact on team work in daily health care practice. Nevertheless, there are various challenges for sustainable implementation of IPE. To identify enablers and barriers of IPE for medical and nursing students as well as to specify impacts of IPE for both professions, the ‘Cooperative academical regional evidence-based Nursing Study in Mecklenburg-Western Pomerania’ (Care-N Study M-V) was conducted. The aim is to explore, how IPE has to be designed and implemented in medical and nursing training programs to optimize students’ impact for IPC.

**Methods:**

A qualitative study was conducted using the Delphi method and included 25 experts. Experts were selected by following inclusion criteria: (a) ability to answer every research question, one question particularly competent, (b) interdisciplinarity, (c) sustainability and (d) status. They were purposely sampled. Recruitment was based on existing collaborations and a web based search.

**Results:**

The experts find more enablers than barriers for IPE between medical and nursing students. Four primary arguments for IPE were mentioned: (1) development and promotion of interprofessional thinking and acting, (2) acquirement of shared knowledge, (3) promotion of beneficial information and knowledge exchange, and (4) promotion of mutual understanding. Major barriers of IPE are the coordination and harmonization of the curricula of the two professions. With respect to the effects of IPE for IPC, experts mentioned possible improvements on (a) patient level and (b) professional level. Experts expect an improved patient-centered care based on better mutual understanding and coordinated cooperation in interprofessional health care teams. To sustainably implement IPE for medical and nursing students, IPE needs endorsement by both, medical and nursing faculties.

**Conclusion:**

In conclusion, IPE promotes interprofessional cooperation between the medical and the nursing profession. Skills in interprofessional communication and roles understanding will be primary preconditions to improve collaborative patient-centered care. The impact of IPE for patients and caregivers as well as for both professions now needs to be more specifically analysed in prospective intervention studies.

## Background

A changing health care system with increasingly complex health needs of patients require innovative and efficient concepts of patient care. These concepts require key competencies, such as effective communication, teamwork and interprofessional collaboration between healthcare professionals [1, 2]. Interprofessional education (IPE), whereby students from several healthcare professions learn and work together [3, 4], has shown a positive impact on team work in daily health care practice [5] and is recommended for training programs of healthcare professionals [6–8]. Several advantages of IPE have been reported: (a) increased mutual respect and trust [9, 10], (b) improved understanding of professional roles and responsibilities [10–13], (c) effective communication [1, 7], (d) increased job satisfaction [11, 13, 14], and (e) positive impact on patient outcomes (e.g. decreased patient’s length of hospital stay and a reduced number of medical errors) [8, 15, 16]. Previous studies have proven that students trained in an IPE approach have better interprofessional collaborative practice competencies compared to students without an IPE-training [8, 15, 17]. This can be attributed to students’ more positive attitudes towards each other, a better understanding about each other’s competencies, the ability to share knowledge and skills, and improved team identity [10, 13, 17]. Nevertheless, there are various challenges for sustainable implementation of IPE including (a) non-coordinated and strictly separate curricula of different health care professions, (b) an insufficient number of specifically qualified teaching staff and (c) limited financial resources of the institutions [9, 12, 18, 19]. As a result, most existing IPE courses are optional and only a few of them are sustainably implemented in the curricula of the health care professionals involved.

In Germany, IPE and research on the impact for interprofessional collaboration (IPC) in routine care is still in its iunfancy. First IPE activities concerned interprofessional communication in hospitals [20], interprofessional seminars in ethics [21], and interprofessional emergency management [22]. To support a sustainable implementation of IPE the GMA Committee - ‘Interprofessional Education for the Health Care Professions’ was founded in 2011. In its position statement, the committee developed recommendations to integrate interprofessional approaches into education for health professions and required continuous evaluation regarding outcomes of IPE [23]. In addition, the Advisory Council on the Assessment of Developments in the Health Care System emphasize in its report “Cooperation and Responsibility” in 2007 positive effects of IPE for IPC including a better mutual understanding and the acquisition of cooperative skills for all professions involved [24]. A better interaction between the different health care professions is a further positive effect that the GMA Committee stated [23].

To specify the impacts of IPE for medical and nursing students and to identify enablers and barriers of IPE, the Cooperative academical regional evidence-based Nursing Study in Mecklenburg-Western Pomerania (Care-N Study M-V) was conducted. A starting point of the study was the development of an academic nursing program [25]. The study evaluated (1) IPE acceptance between medical and nursing students, and four further research dimensions: (2) further development of academic nursing training, (3) identification of the task fields for graduates with bachelor degrees or master degrees in nursing, (4) specification of learning contents for academic bachelor and master training programs and (5) implications for health politics. The research dimensions comprised 25 research questions. A detailed study design and selected preliminary results were published elsewhere [25, 26].

Research dimension (1) IPE between medical and nursing students addressed the following research questions: (a) what are the enablers and barriers of IPE for nursing and medical students? (b) what are the expectations for the future impact of IPC between both professions?. The aim of the investigation is to explore, how IPE should be designed and implemented in medical and nursing training programs to optimize students’ impact for IPC in routine care.

## Methods

### Design

The present analyses are based on data derived from the Care-N Study M-V, which was a qualitative study using the Delphi method (type: aggregation of ideas) and consisted of two qualitative semi-structured mailed questionnaires, and a group discussion. The details of the study have been described elsewhere [25].

### Participants

To guide recruitment of experts for the study, inclusion criteria based on Häder 2009 were defined: (a) each expert is able to answer every research question, one question particularly competent, (b) interdisciplinarity, (c) sustainability and (d) status [27]. To be able to cover a wide range of ideas several disciplines were involved (interdisciplinarity). The sustainable implementation of the study results was supported by experts from different stakeholders associations and politics (sustainability) [28]. It was expected that expert answers will depend on their hierarchy level. Thereby, it could not readily assume that experts in higher positions have all the necessary expertise [29]. Therefore, experts representing various hierarchy levels were included (status of the person).

Participants were purposely sampled. Recruitment was based on existing collaborations and a web based search. A fixed order for enquiry of experts was defined. When a ‘first’ requested expert declined to participate, the ‘second’ expert from the respective list was contacted. This process was repeated until for every research question an adequate expert could be recruited. The first contact was made by telephone: the study was described and the interest for participation was solicited. When the feedback was positive, experts received written study information detailing study aims, methods, data management and a written informed consent. When the written informed consent was completed, signed, and returned to the study center, experts were enrolled in the Care-N Study M-V [25].

Nine experts rejected their participation. Reasons for non-participation provided included: (a) lack of time, (b) stay abroad during the data collection phase of the study, and too short-term request. Over the course of the study none of the experts dropped out.

**Fig. 1:**
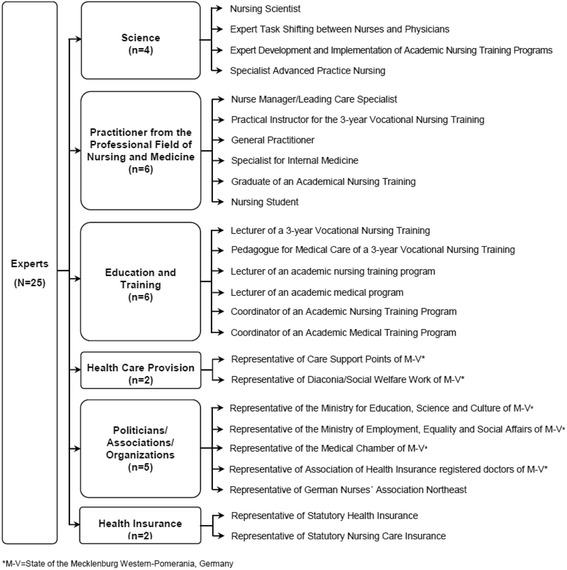
Experts of the Care-N Study M-V

Overall, 25 experts were enrolled in the Care-N Study M-V. Each expert was categorized to one of following six professional areas: (1) Science, (2) Practitioner from the Professional Fields of Nursing and Medicine, (3) Education and Training, (4) Health Care Provision, (5) Politicians, Associations, Organizations and (6) Health Insurance (see Fig. 1).

Experts were invited to participated in all data collection rounds and to answer all questions. They decide by themselves, in which data collection round they want to participate and which questions they answer. The distribution was: (a) first qualitative mailed questionnaire *n* = 13, (b) second qualitative mailed questionnaire *n* = 14 and (c) group discussion *n* = 9). At least, each expert was involved in one of the three data collection rounds. Most of them participate in two of the three data collection rounds. Detailed description can be found by Dreier et al. 2016, doi: 10.1016/j.zefq.2016.03.003.

The majority of experts were female (*n* = 16). Of them, 13 experts (52%) participated in the first mailed questionnaire, 14 (56%) in the second and nine (36%) in the focus group discussion. All 25 respondents participated at least in one of the three data collections [25].

### Data collection

The three data collection rounds were conducted in intervals of 3 months between June 2013 and April 2014. The first qualitative mailed questionnaire consisted of 25 semi-structured research questions. Its completion took 30–50 min. Of this, five research questions addressed IPE. Results of the first qualitative semi-structured interviews with 13 experts were sent to all experts as a written summary. This summary is a key feature of a Delphi method [27]. And was the basis for the second mailed questionnaire with 14 experts, which consisted of 17 semi-structured research questions (two of those specifically addressing IPE) of the overall 25 research questions. Experts completed and specified their answers based on the results of the first semi-structured mailed questionnaire. The completion took 20–35 min. A written summary with the results were sent to all experts by mail. Results were the basis for the group discussion to clarify specific aspects of both semi-structured questionnaires.

The focus group discussion was conducted with nine experts and comprised five semi-structured research questions. It took approximately 3 h. One team member was the interviewer. The focus group discussion took place in the study center of the Institute for Community Medicine, University Medicine Greifswald, Mecklenburg-Western Pomerania, Germany. All experts received the results as a written summary by mail. Data saturation was evident in the way that no new information was forthcoming during the focus group interviews for the 20 of overall 25 research questions.

The survey instrument for the two semi-structured questionnaires was digitized using TeleForm® (Electric Paper Information Systems GmbH Lüneburg Germany, version 10.2). The completed questionnaires were scanned and verified in TeleForm®. For analysis, the data were documented in a word-data base and transferred to the software MAXQDA, Version 10 (VERBI GmbH, Berlin). The focus group discussion was audio recorded and transcribed with the f4transkript software (dr. dresing & pehl GmbH, Marburg).

### Data analysis

To analyze both qualitative semi-structured interviews and the group discussion, a qualitative content analysis as suggested by Kuckartz et al. [30, 31] using the software MAXQDA was conducted. Analysis steps were: (1) Initiating textual work (highlight important text passages, writing memos), (2) development of thematic major categories, (3) coding of the questionnaires and group discussion transcripts by the thematic major categories, (4) assort text passage with the same thematic major categories, (5) inductive development of sub-categories based on the questionnaires and group discussion, (6) coding of the complete text material by the differentiated category system (with thematic major categories and sub-categories) and (7) category based analysis and result presentation. This analysis structures qualitative data into an order and by frequencies [30].

Two study team members (SH, ADW) coded the interviews and the group discussion according to the consensual coding approach [30]. First, both team members coded separately. Subsequently, both resulting category systems were compared for similarities and differences. In case of differences, the codes were discussed and modified if both coders agreed. This process caused an extension of the category system. Finally, a system with categories, sub-categories and codes based on the code systems of both coders was developed [25].

## Results

### Enablers and barriers for IPE

Experts stated more enablers then barriers for IPE for medical and nursing students (see Table 1). Overall, four primary arguments for IPE in medical and nursing training programs were mentioned: (1) development and promotion of interprofessional thinking and acting (2) acquirement of shared knowledge, (3) promotion of beneficial information and knowledge exchange, and (4) promotion of mutual understanding.

**Table 1:** Enablers and barriers of IPE for medical and nursing students

Enablers
Development and promotion of interprofessional thinking and acting patient centered care Acquisition of shared knowledge Promotion of information and knowledge exchange Promotion of mutual understanding mutual acceptance respect for each other reduction of hierarchies Prerequisites for successful IPC specific skills and knowledge communication specific roles and tasks of medical and nursing profession ability to put oneself in the other profession’s perspective
Barriers
Standardization of learning content levels Different levels of knowledge Chronological harmonization of medical and nursing curricula Organization of IPE lectures personal resources time resources financial resources Low mutual respect between medical and nursing students and resulting limited willingness for IPE Low appreciation of medical students towards nursing students Capacity-legal issues^a^

^a^ In Germany, the number of medical students, that a University hast to accept, is determined by a formula that contains the faculty’s total teaching hours and class sizes. As a consequence, any engagement of faculty members in additional teaching formats provides an argument for additional medical students to enforce their acceptance legally

*‘Interprofessional education for medicine and nursing students promotes the recognition and understanding of interdisciplinary correlations, the competence for interprofessional acting […] and the clinical expertise.’ (FB_1114: 137–137).*

One argument for IPE *‘[…] is the mutual benefit to investigate themes from medical’s and nursing’s perspectives.’ (FB_1119: 88–88).*

According to experts’ opinions a mutual understanding particularly supports the acceptance and respect for each other in caring for patients in daily work. The reduction of hierarchies is an additional positive side effect.

IPE *‘[…] improves the acceptance and thus the mutual understanding of both professions.’ (FB_1113: 59–59).*

*‘Interprofessional education can dismantle hierarchies between both professions.’ (FB_1115: 92–92).*

To further develop IPC, experts mentioned three main skills or competences, which need to be taught by IPE for medical and nursing students: (1) interprofessional communication, (2) profession specific roles and tasks as well as (3) put oneself in the other profession’s perspective. In consequence, experts concluded that these are the main preconditions for improving IPC in the long run to address the overall aim of optimization of needs-oriented and patient centered care.

*‘IPE promotes skills and competences to work effectively in an interprofessional team. Improved communication enhances coordinated interprofessional collaborative practice.’ (FB_1115: 92–92).*

*‘The recognition and understanding of interdisciplinary relation help to identify threatening or current health problems.’ (FB_1114: 137–137).*

Hence, for any broader rollout of IPE, various barriers must be overcome (see Table 1).

The experts emphasized (1) standardization of learning content levels, (2) different levels of knowledge and (3) the chronological harmonization of medical and nursing curricula as the major challenges of IPE.

*‘The different levels of knowledge should be noted, training programs have to be synchronized.’ (FB_1115: 95).*

*‘Width and depth of the medical knowledge differ from the nursing knowledge.’ (FB_1112: 66).*

Furthermore, implementing IPE for all students in medical and nursing curricula may cause capacity-legal challenges.

IPE *’[…] leads to capacity-legal problems, because of the restricted acceptance of medical students at medical faculties.’ (FB_1115: 95–95).*

This also includes the organization of IPE lectures, which requires personnel, time and financial resources. To overcome existing challenges of IPE for medical and nursing students, experts stated, that the willingness of both students groups and the mutual respect are the most important prerequisites for successful implementation.

*’The feasibility of IPE is associated with a high effort which needs essential personal, time and financial resources.’ (FB_2114: 51).*

### Expectations for future IPC in routine care

**Fig. 2:**
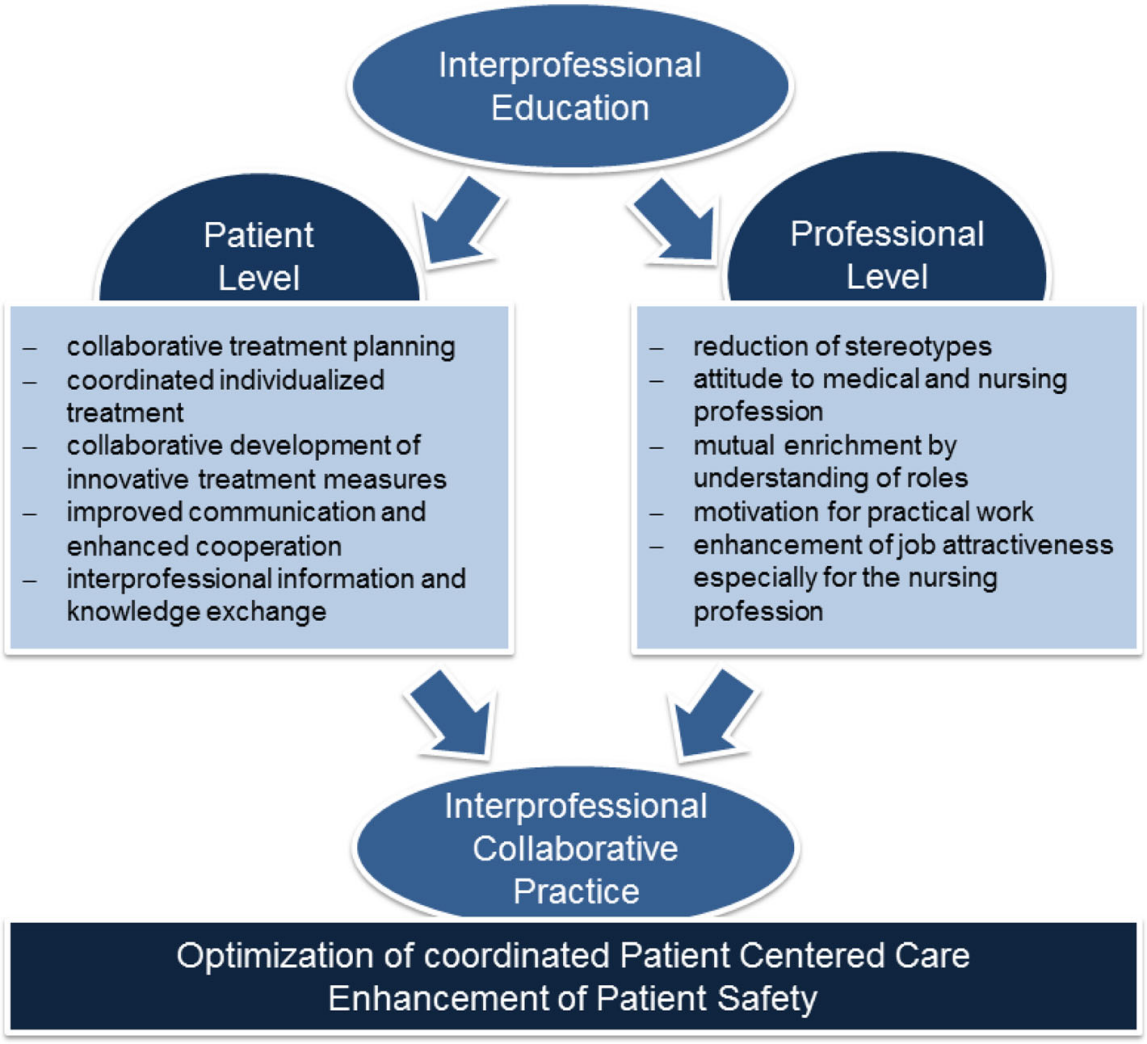
Effects of IPE for medical and nursing students for future interprofessional collaborative practice

Judging the effects of IPE for medical and nursing students for future IPC to experts differentiated the (a) patient level and (b) professional level (see Fig. 2).

On the patients’ level, experts mentioned possible improvements in following areas: (a) collaborative treatment planning (b) coordinated treatment and implementing individualized health care interventions, (c) collaborative development of innovative treatment measures, (d) improved communication and enhanced cooperation during the care process, and (d) continuous interprofessional information and knowledge exchange to be able to adapt a care plan quickly when necessary.

*‘I expect an improved collaborative treatment planning as well as an enhanced integrated care of patients.’ (FB_1111: 73–73)*

*‘[…] the shared development and implementation of new concepts of patient care.’ (FB_1101: 122)*

*‘[…] an improved competence to work as a team in the daily health care practice in consideration of patients’ interest.’ (FB_1119: 107–107)*

Based on this further development of IPC, experts expect changes on the professional level including (a) reduction of stereotypes, (b) improvement of mutual attitude toward the medical and the nursing profession and, (c) mutual appreciation by understanding of roles (see Fig. 2).

*‘I expect a reduction of stereotyped behaviors’ as well as improved confidence and understanding in working with the other professional group.’ (FB_1103: 89–89)*

*‘Everybody has an improved understanding of professional responsibilities of all team members.’ (FB_1101: 121)*

This might also enhance motivation for practical work in both professions as well as increase job attractiveness especially for the nursing profession (see Fig. 2).

*‘[…] increase of motivation for the practical work.’ (FB_1103: 89)*

*‘[…] improvement of job attractiveness for the nursing profession.’ (FB_1115: 115)*

In consequence, experts assume an optimization of patient centered care by coordinated collaboration, which results in an enhancement to patient safety (see Fig. 2).

*‘I expect a coordinated procedure of both professions in patient care as well as the development of new cooperation models.’ (FB_1114: 165–165)*

*‘[…] patient safety will be improved’ (FB_1101: 120–122)*

### Implications for sustainable implementation of IPE in medical and nursing curricula

**Fig. 3:**
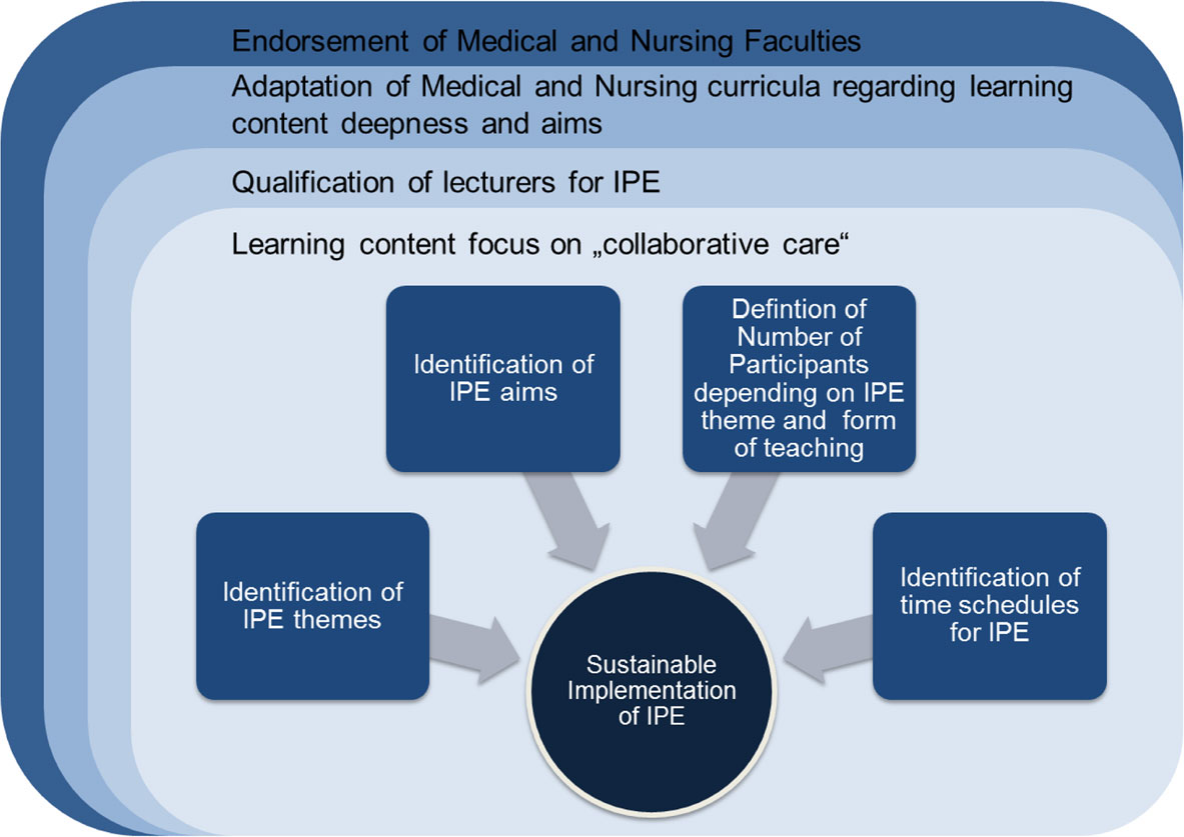
Influencing factors for sustainable implementation of IPE in medical and nursing curricula

To achieve possible positive effects on IPC, a sustainable implementation of IPE in the education of medical and nursing students is required. Experts stated four primary influencing factors, which need to be considered: (a) the commitment of medical and nursing faculties for IPE, (b) the necessity to synchronize currently existing medical and nursing curricula with respect to comparable learning content deepness and learning aims, (c) the qualification of lecturers to adequately prepare them for teaching in an IPE approach and, (d) prioritize IPE learning contents with regard to the added value for patients and their caregivers of interprofessional collaborative practice (see Fig. 3).

‘*The implementation of IPE depends on overcome of different structural factors of the both educational institutions (vocational schools vs. medical faculties of universities).’ (Fragebögen\FB_2103: 45 - 46)*

*‘Different designs of both training programs - with different depths and scopes of individual fields - are taken into account.’ (FB_2115: 40–40)*

*‘It could be critical if lectures are not adequately prepared for interprofessional teaching.’ (FB_2103: 49–49)*

The experts point out, that it is necessary to comprehensively define IPE themes for both professions followed by the identification of learning aims, the determination of the optimum number of participants depending on IPE theme and teaching format as well as to define the optimal time schedule to implement IPE lectures into both training programs (Fig. 3).

*‘A possible disadvantage arises when curricular decisions and reforms include needs and targets for only one (and not for both) professional group(s).’ (FB_1104: 100–100)*

*It should be asked at which interfaces the mutual understanding and the collaborative practice will provide the best possible health benefit to patients. Here interprofessional education should take place. It is important to learn in competence-focused (not fact-focused) useful learning settings. (FB_2119: 29–29)*

## Discussion

The Care-N study M-V aimed to identify enablers, barriers for and impact of IPE on future IPC for the medical and the nursing profession. Overall, the experts surveyed mentioned more enablers then barriers for IPE. Expected impacts for future IPC were stated on patient and professional level to make a contribution toward optimizing coordinated patient centered care and to enhance patient safety in the future.

The results showed that IPE for medical and nursing students can encourage positive mutual attitudes, better understanding of professional roles in caring for patients and their caregivers as well as improved information and knowledge exchange to cooperate during their daily practical work. Previous studies reported similar results and emphasized that students*’* improved attitude towards teamwork increased mutual respect and understanding between different groups of health care professionals [10, 32]. Furthermore, IPE provides opportunities to develop interprofessional communication skills [33, 34], and to exercise how to cooperate in an interprofessional team [7]. The acquirement of profession-specific roles during patient treatment is one of the primary enablers of IPE in the Care-N Study. This result agrees with findings of previous studies showing that IPE prepares students to better understand their own professional identity as well as their roles and perspectives on patients and their caregivers [19, 35, 36].

Furthermore, IPE enables students to learn and exercise interprofessional communication. By improving knowledge about their own roles and responsibilities as well as the roles of the other profession an improvement for interprofessional team working can be expected [36, 37]. Finch stated that students can also be better prepared for performing roles traditionally taken over by the other profession [38]. Due to the fact, that tasks and roles of medical and nursing professionals increasingly overlap [39], and both healthcare professions have expanded their roles and responsibilities [40]. IPE might provide the opportunity to better prepare students to taking over the future extended roles and responsibilities already during their training programs.

The Care-N Study M-V experts identify several barriers against a sustainable implementation of IPE in the medical and nursing curricula. These include difficulties in coordinating the two programs. This finding is consistent with previous studies, which showed that program structures and timetables of both curricula are quite different. This has been reinforced by different regulations for professional training programs and the fact that the programs are offered by different providers (university, university of applied science, vocational school, public and private sector etc.) [8, 12, 18, 23].

Nevertheless, from the experts’ points of view there are various positive impacts for IPC on patient level in the future including an improved patient-centered care and improved coordinated cooperation in interprofessional health care teams. Previous studies showing that an effective cooperation between nursing and medical professionals improves efficiency and quality of patient care as well as patient safety and patient satisfaction. The University of Colorado integrated patient safety into medical and nursing school curricula and tested models for interprofessional student involvement in clinical improvement [41]. As a result medical and nursing students improved the efficiency of discharge processes, safety of patient transfers from intensive care, and care for bedsores [41]. In their evaluation of an interprofessional student clinical program, Lawrence et al. could show that patients reported high levels of satisfaction with the patient care team and the facility quality [42]. In particular results of the items students ‘listen to you’, ‘take enough time with you’, ‘explain what you want to know’, ‘answer your questions’ described high levels of patient satisfaction [42].

To achieve these positive impacts for IPC, IPE must be widely implemented in both professions’ curricula. One important precondition is, that existing curricula need to be opened and revised for this learning approach. Experts of the Care-N Study stated that IPE should focus on collaborative care to maximize the value for both patients and caregivers. Liang et al. examined outcomes of Neighborhood Health Screening (NHS), an in-home service provided by medical and nursing undergraduate students for 355 patients in a low-income neighborhood [43]. Overall, 240 medical and 34 nursing students were involved in management of chronic diseases, particularly hypertension. NHS had a positive impact on hypertension management. Demonstrably the rates of treatment for blood pressure (63 to 93%) and control of blood pressure (27 to 73%) could be improved already over 1 year [43].

The Care-N Study M-V has several limitations. First, the use of purposive sampling can be highly prone to selection bias. The definition of clear inclusion criteria should antagonize this potential bias. Second, the purposive sampling focuses on particular characteristics of an expert population enabling them to best answer the research questions. Thus, generalization to the whole population is limited.

## Conclusion

The Care-N Study M-V describes enablers, barriers and impact of IPE for medical and nursing students. IPE promotes interprofessional cooperation between both professions and enhance mutual respect and understanding. For future IPC in routine care it can be expected that skills in interprofessional communication and roles understanding will be primary preconditions to improve collaborative patient-centered care. A sustainable implementation of IPE programs faces various barriers, including the coordination of IPE with the medical and nursing core curricula. In consequence, it is necessary to specify overlapping tasks fields to comprehensively identify IPE themes for both professions. Furthermore, the actual impact on patients health and on caregiver burden as well as on the development of both professions need to be studied in controlled prospective designs in the future.

## Abbrevations

Care-N Study M-V: Cooperative academical regional evidence-based Nursing Study in Mecklenburg-Western Pomerania
GMA: Society for Medical Education (Gesellschaft für Medizinische Ausbildung)
IPC: Interprofessional collaboration
IPE: Interprofessional education
NHS: Neighborhood health screening
WHO: World Health Organization
